# Ageing- and dementia-friendly design: theory and evidence from cognitive psychology, neuropsychology and environmental psychology can contribute to design guidelines that minimise spatial disorientation

**DOI:** 10.1007/s10339-021-01031-8

**Published:** 2021-05-28

**Authors:** Jan M. Wiener, Francesca Pazzaglia

**Affiliations:** 1 Department of Psychology, Bournemouth University, Poole, UK; 2 Ageing and Dementia Research Centre, Bournemouth University, Poole, UK; 3 Department of General Psychology, University of Padova, Padova, Italy; 4 Inter-University Research Centre in Environmental Psychology (CIRPA), Rome, Italy

**Keywords:** Spatial orientation, Wayfinding, Ageing, Dementia, Dementia-friendly design, Environments

## Abstract

Many older people, both with and without dementia, eventually move from their familiar home environments into unfamiliar surroundings, such as sheltered housing or care homes. Age-related declines in wayfinding skills can make it difficult to learn to navigate in these new, unfamiliar environments. To facilitate the transition to their new accommodation, it is therefore important to develop retirement complexes and care homes specifically designed to reduce the wayfinding difficulties of older people and those with Alzheimer’s disease (AD). Residential complexes that are designed to support spatial orientation and that compensate for impaired navigation abilities would make it easier for people with dementia to adapt to their new living environment. This would improve the independence, quality of life and well-being of residents, and reduce the caregivers’ workload. Based on these premises, this opinion paper considers how evidence from cognitive psychology, neuropsychology and environmental psychology can contribute to ageing- and dementia-friendly design with a view to minimising spatial disorientation. After an introduction of the cognitive mechanisms and processes involved in spatial navigation, and the changes that occur in typical and atypical ageing, research from the field of environmental psychology is considered, highlighting design factors likely to facilitate (or impair) indoor wayfinding in complex buildings. Finally, psychological theories and design knowledge are combined to suggest ageing- and dementia-friendly design guidelines that aim to minimise spatial disorientation by focusing on residual navigation skills.

## Introduction

The lifespan of the world’s population is increasing, and the proportion of people over 60 years old is predicted to rise from 12% in 2015 to 22% in 2050 (World Health Organisation (2015)). People are susceptible to decline in their cognitive abilities as they age (Salthouse 2010), and 5–8% of the general population aged 60 and over will develop dementia at some point (World Health Organisation 2017). Worldwide, there are currently approximately 50 million people living with dementia, and almost 10 million new diagnoses are established every year (World Health Organisation 2019). Among the different types of dementia, Alzheimer's disease (AD) is the most prevalent form, accounting for 60–70% of cases (World Health Organisation 2019).

Both typical ageing and dementia result in marked declines of orientation and navigational abilities (Benke et al. 2014; Lester et al. 2017). Spatial orientation and navigational skills start deteriorating relatively early in the ageing process, even when there are no apparent deficiencies in other cognitive abilities (Harris and Wolbers 2012; Moffat 2009). This gradual loss of navigational skills is even more evident in individuals with Mild Cognitive Impairment (MCI) (Fasano et al. 2018; Mitolo et al. 2013) or AD, of which disorientation is typically one of the first symptoms (Pai and Jacobs 2004; Serino et al. 2015), and in unfamiliar environments. Research on typical ageing has demonstrated that older adults show similar navigation performance to young adults in spatial tasks in familiar environments (e.g. Kirasic 1991; Lopez et al. 2018); for exceptions see (Muffato et al. 2020), whereas the decline in orientation and wayfinding skills makes it difficult for older adults to navigate and learn new, unfamiliar environments. This is problematic as a considerable proportion of older people with and without dementia eventually move from their familiar home environments into unfamiliar surroundings, such as sheltered housing or care homes. In 2011, the proportion of people in the EU who were aged 65–84 years and living in an institutional household (health care institutions or institutions for retired or elderly persons) was 1.7%; among those aged 85 years and over, this share reached 12.6% (EUROSTAT 2019). In 2014, already 38% of the people with dementia in the UK were living in residential care or nursing homes (Prince et al. 2014).

Designing retirement complexes and care homes with people whose navigational skills are declining in mind would make the transition to their new environment easier for people with AD (O’Malley et al. 2017). To enable the layouts of larger environments—such as care homes, retirement complexes, or even hospitals—to be learned with relative ease, these environments need to be designed so that they facilitate spatial orientation and compensate for declining navigation abilities. This would enable residents to adapt more easily to their new accommodation, improve their quality of life and well-being, enable them to retain a greater degree of independence, and reduce their caregivers’ workload (Marquardt and Schmieg 2009).

There are multiple design guidelines and auditing tools that address the ‘dementia-friendliness’ of an environment (O’Malley et al. 2017): the Dementia Audit Tool (DAT) (Dementia Services Development Centre 2011); the EVOLVE design toolkit (Orrell et al. 2013); the Enhancing the Healing Environment (EHE) assessment tool (The King’s Fund 2013); the Environmental Audit Tool (EAT) (Fleming 2011); and the NHS Scotland Wayfinding document (Health Facilities Scotland 2007), the only one completely dedicated to wayfinding in healthcare facilities, but not specifically focused on residences for aged people. While these guidelines provide advice on how to improve the design of care environments to support people with AD and other types of dementia, only few design suggestions specifically focus on alleviating disorientation and aiding wayfinding, and those that do are often not well supported by neuropsychological theories of navigation (O’Malley et al. 2017). In this article, we argue that (neuropsychological theories of orientation and navigation, backed by experimental evidence, could—and should—play a major part in improving the design of care environments.

### Goals

In this paper, we integrate knowledge from different disciplines, including cognitive psychology, neuropsychology, environmental psychology, and architectural design. Our goal is to provide evidence-based guidelines that specifically reduce spatial disorientation and support wayfinding that can be applied in designing and reconstructing residential care and nursing homes for aged people. Given that we (1) consider literature from different disciplines and (2) discuss how findings presented in a different context and for a different purpose can inform ageing- and dementia-friendly design, we decided against carrying out a systematic literature review. Instead, we present an opinion paper in which we argue that effective ageing- and dementia-friendly design should be informed by evidence from a variety of disciplines and research areas. In particular, we here focus on the contributions that (neuro-)psychology and environmental psychology can make to the development of improved design guidelines that minimise spatial disorientation.

It should be noted at this point that we will focus particularly on AD, although we refer to other forms of dementia where necessary throughout the paper. This is for a number of reasons: First, AD is by far the most prevalent form of dementia, affecting ~2/3 of all people living with dementia (World Health Organisation 2019). Second, AD affects spatial orientation abilities more dramatically than other forms of dementia because of the overlap of brain areas affected by AD and those associated with navigation and orientation behaviour (Pengas et al. 2010). Third, our aim is to provide evidence-based suggestions to improve dementia-friendly design guidelines and AD is by far the most frequently researched form of dementia (World Health Organisation 2019), particularly with respect to navigation and orientation abilities (Serino et al. 2015). However, given the more general decline of orientation and navigation abilities in older age (as described in more detail in "Spatial reference frames"), our design suggestion could improve navigation and wayfinding also in healthy older adults and people affected by other forms of dementia.

We first discuss cognitive mechanisms and processes involved in spatial navigation, and discuss how navigation and wayfinding abilities are affected by typical and atypical ageing. We then look at research from the field of environmental psychology to focus on design factors likely to facilitate (or impair) indoor wayfinding in complex public buildings, such as hospitals, museums, department stores, and so on. Finally, we combine cognitive and neuropsychological theories and design know-how to suggest improved ageing- and dementia-friendly design guidelines with a view to minimising spatial disorientation in older adults with and without cognitive impairments by targeting residual navigation abilities. Our aim is to provide generic design guidelines or principles. Such design principles can then be translated into bespoke solutions for specific environments.

## Navigation: an overview

### User-environment interactions in spatial navigation and wayfinding

Wayfinding, the coordinated and goal-directed movement through the environment (Montello et al. 2005), is defined as the process of determining and navigating a route from an origin to a destination (Golledge et al. 1987). Wayfinding is a complex, multicomponent ability susceptible to broad individual differences (Hegarty and Waller 2005) and affected by emotions and motivation. For instance, spatial anxiety has a detrimental effect on wayfinding performance, particularly in complex navigation tasks (Srinivas 2011), whereas a sense of spatial self-efficacy and taking pleasure in exploring unfamiliar environments positively correlate with wayfinding performance (Pazzaglia et al. 2018a, b). Good wayfinding abilities (derived from the interaction between individual and environmental factors) are an important source of quality of life and autonomy. Emotions became still more relevant in atypical ageing when people with dementia interact with non-familiar environment. Several studies demonstrated that the role of the environment is of paramount importance in reducing stress and promoting quality of life. In a review on the impact of the design of the built environments in people with dementia, Marquardt et al. (2014) found that small-scale environments, an environmental aspect strictly related to spatial navigation (Marquardt and Schmieg 2009), are associated with the reduction in dysfunctional behaviour and improved well-being including lessened depressive symptoms and improved mood and quality of life.

The ability to plan a route, move towards and reach a destination derives from the interaction between individual skills and environmental factors (Pazzaglia et al. 2017). Numerous interactive models of navigation and wayfinding have been proposed. In the following, we will be reviewing interactive models of navigation and navigation studies. Even though the aim of this paper is to provide design suggestions for care and residential environments, i.e. indoor environments, people employ the same navigation mechanisms in indoor and outdoor environments. We therefore cite research that made use of both types of environments.

Carpman and Grant (2002) analysed indoor wayfinding behaviour in terms of systems in which combinations of behavioural factors (cognitive variables and individual differences), building design, and operational elements (e.g. indoor signage, you-are-here maps, etc.) interact to create an environment that may be more or less easy to navigate. Carlson et al. (2010) suggested considering indoor wayfinding complexity in terms of environmental variables (a building’s features), people’s spatial representations or cognitive maps (a sort of mental image of the environment) (Tolman 1948), and personal factors (an individual’s spatial abilities and strategies). The way in which these elements interact can be analysed in terms of: correspondence (between a building and a cognitive map); compatibility (between a building and an individual’s strategies); and completeness (between a cognitive map and an individual’s spatial abilities and strategies). Analysing these components together can shed light on the navigation problems posed by a given building.

A similar interactive approach is also taken by ecological gerontology models that explain the relationship between individual characteristics and environments in terms of a person-environment fit (Kahana 1982), where individuals’ ability to express their full potential depends on their compatibility with their environment. The ability to cope with the environment may diminish as people become more vulnerable due to ageing (Schröder-Butterfill and Marianti 2006). Such vulnerabilities result in people becoming increasingly dependent on their environment, as envisaged by the Environmental Docility Hypothesis (Lawton 1977; Lawton and Simon 1968; Fornara et al. 2019). This also explains why individual differences in wayfinding behaviour (Wolbers and Hegarty 2010) should be analysed bearing the environmental variables that facilitate (or inhibit) navigation in mind (Devlin 2014). For these reasons, we will analyse cognitive aspects and other individual factors involved in navigation in the following section before we review environmental variables that affect navigation. Evidence from these individual and environmental factors will be used to motivate the design guidelines listed in the last section of the paper.

### Cognitive aspects of navigation and wayfinding

Navigating is a complex task that relies on a number of sensory and cognitive processes, and on enduring as well as transient spatial representations (Wolbers and Hegarty 2010). Sensory inputs from visual and auditory receptors provide information on the way the surroundings look and sound. Visual input, vestibular receptors and proprioceptors provide information about how people move through space. All these inputs are processed and integrated to enable people to locate their position and to orient themselves, and to plan routes to intended destinations (for an overview, see (Wolbers and Hegarty 2010)).

Navigation is based on two principal systems: *path integration*; and *landmark-based navigation* (Zhao and Warren 2015). Path integration refers to the process of updating information of perceived self-motion to keep track of position and orientation during travel (Wang and Spelke 2002). Landmark-based navigation relies on visual landmarks and other environmental information for homing, reorientation, and wayfinding (Trullier et al. 1997). The path integration and landmark-based navigation systems work in parallel in everyday navigation and both systems are affected by ageing. In this paper, we focus primarily on the landmark-based navigation system, which relies on environmental cues (landmarks) to help people find their way, and is therefore more amenable to improvement by means of appropriate design choices.

### Landmark-based navigation

#### Landmark-based navigation

Crucially depends on the recognition and use of landmarks and other environmental features to ascertain where one is in an environment, and guide steps in the right direction (Krukar et al. 2017). A landmark is typically defined as any object or feature of an environment that is easy to see and recognisable—especially one that helps to establish position (Richter and Winter 2014). To be useful for the purposes of orientation and wayfinding, landmarks must have a number of properties (Nothegger et al. 2004; Raubal and Winter 2002; Stankiewicz and Kalia 2007a).

#### Uniqueness

Landmarks must be unique to aid successful navigation (Stankiewicz and Kalia 2007a; Viaene et al. 2014; Strickrodt et al. 1936). Common objects or environmental cues that we can encounter in different places can be confusing and disorienting because they cannot be used to unambiguously identify a given location.

#### Saliency

Landmarks must be salient, meaning that they need to stand out against the rest of the environment, so they will be easily noticed and recognised when we return to the same place (Stankiewicz and Kalia 2007a). In outdoor navigation, for example, the main features of objects chosen as landmarks are their strong contrast with their setting (Ishikawa and Nakamura 2012). This contrast may concern their colour (buildings with more saturated colours are chosen more frequently than those with less saturated colours), size (bigger is better), or style (in a traditional neighbourhood, a modern building will have more appeal).

#### Persistence

Landmarks must remain in the same location, so that they can be found in the same place when returning (Stankiewicz and Kalia 2007a).

#### Informativeness

Landmarks can be informative in two ways. First, they can provide information about position, thus enabling one to orient in the surroundings. Second, they provide navigational information that enables to move in the right direction towards the destination (Stankiewicz and Kalia 2007a). This is best demonstrated by the use of landmarks when navigating a route: they can serve as associative cues if a movement in a particular direction is associated with a landmark (‘turn right at the church’), or as beacons if they can be seen from the decision point (see below) and turning towards them brings the navigator closer to the goal (‘turn towards the church’) (Waller and Lippa 2007; Wiener et al. 2013; Chan et al. 2012).

#### Nameable

How easily nameable a landmark is, affects how likely it is to be selected as a landmark (Klippel and Winter 2005). For example, when giving directions, nameable landmarks are preferred over non-nameable landmarks (Stankiewicz and Kalia 2007b).

#### Location

For objects or environmental features to be used as landmarks, they need to be in a navigationally relevant location. Typically, an object is chosen as a landmark when it is located at, or can be seen from, a point where we need to make a decision (Jansen-Osmann 2002). Objects or environmental features located in between decision points can still serve as landmarks, however, by reassuring navigators that they are going the right way (Anacta et al. 2017). This has the potential to reduce spatial anxiety in people with orientation problems as well as facilitating navigation.

#### Spatial reference frames

When navigating, people can encode spatial information, such as the positions of landmarks, in either an egocentric (body-centred) or allocentric (world-centred) frame of reference, or coordinate system (Ekstrom et al. 2014; Wolbers and Wiener 2014). This distinction is important because egocentric and allocentric representations, and the corresponding navigation strategies, are supported by different brain areas (Hartley et al. 2003), some of which are affected more than others by typical and atypical ageing (Lester et al. 2017; Lithfous et al. 2013). These age-related changes will be discussed in more detail in the next section.

Egocentric strategies are often also referred to as ‘route’ or ‘response’ strategies (Lawton 1994, 1996; Tversky 2003). They are most commonly used in familiar environments, where a well-known route can be replicated using a series of stimulus–response associations (Strickrodt et al. 1936). Specifically, the navigator associates a specific navigational or directional response with a particular landmark stimulus, e.g., ‘turn left at the church’. Route memories consisting of a series of stimulus–response associations are rigid, inflexible and unidirectional, i.e. they only allow for navigation from a starting point to a destination.

Allocentric strategies are also known as ‘place’ or ‘orientation’ strategies (Lawton 1994), and rely on spatial representations that are often described as ‘cognitive maps’ (Tolman 1948). Unlike egocentric representations, allocentric representations are independent of an individual’s position in the environment (Wolbers and Wiener 2014). Instead, they are based on the spatial relationships between landmarks and other navigational features, such as buildings or road signs. Allocentric navigation strategies rely on the ability to mentally manipulate cognitive maps, to make sense of them from different viewpoints, and then plan a route from one location to another (Montello et al. 1998). Allocentric strategies are more flexible than egocentric ones, allowing us to plan and navigate different routes between places, and to identify shortcuts through less familiar environments.

## How does ageing affect navigation abilities?

### Healthy ageing

Cognitive ageing is associated with a decline in route learning ability (Head and Isom 2010; Hilton et al. 2021) i.e., older adults take longer to learn novel routes (O'Malley et al. 2018). Zhong and Moffat (2016) argue that ageing affects landmark-direction associations (such as ‘turn left at the church’), a crucial component of route knowledge. Typically ageing adults also show deficits in knowledge of the sequence in which they encountered landmarks along a route (Head and Isom 2010; Hilton et al. 2021). However, if healthily ageing older adults are given sufficient time to learn routes successfully, their knowledge about the landmark-direction associations is as good as that of younger adults (Hilton et al. 2021). Their knowledge about the sequence in which landmarks were encountered on the other hand remains impaired (Hilton et al. 2021). In terms of the underlying neuronal circuits, route learning has been associated with activation of the caudate nucleus (Hartley et al. 2003) and with caudate volume (Head and Isom 2010). The caudate undergoes age-related neurodegenerative changes (Betts et al. 2016) which may explain the route learning impairments described.

Healthy older adults also show deficits in using allocentric navigation strategies. Specifically, healthy older adults take longer to form cognitive maps and, in addition to their difficulty with encoding spatial information, their memory retrieval is also affected by cognitive ageing (Iaria et al. 2009). This can result in older adults mistaking unfamiliar places for familiar ones (Vieweg et al. 2015). Even after learning a novel environment, older adults are less efficient than younger adults in using cognitive maps to plan new routes through an environment (Iaria et al. 2009; Harris and Wolbers 2014; Liu 2011). Allocentric navigation is linked to the entorhinal-hippocampal circuits in the brain (O'Keefe and Nadel 1978). These brain areas are among the first to deteriorate during typical ageing (Moffat 2009), which may explain why allocentric navigation abilities decline in older age.

Finally, healthy older adults also tend to prefer different navigation strategies from younger adults, for an overview, see (Lester et al. 2017). Specifically, older adults find allocentric navigation strategies more difficult to use than egocentric strategies (Wiener et al. 2013; Head and Isom 2010; Iaria et al. 2009) and, given the opportunity to choose, they prefer to use the latter (Rodgers et al. 2012). Successful navigation may also demand switching between navigation strategies or reference frames, and older people appear to have particular problems with switching from an egocentric to an allocentric strategy, while switching from an allocentric to an egocentric strategy is easier (Harris and Wolbers 2012, 2014).

### Atypical ageing

To date, most studies investigating the effects of atypical ageing on navigation abilities have focused on AD and MCI (amnestic MCI is often considered prodromal to AD) (Pai and Jacobs 2004; Serino et al. 2014, 2015; Lithfous et al. 2013; Kunz et al. 2015; Kalova et al. 2005; Cushman et al. 2008; Mokrisova et al. 2016). Our current understanding of how other, less prevalent forms of dementia affect navigation is limited. However, some studies suggest that spatial memory and navigational abilities are less affected in certain types of dementia, including semantic dementia, frontotemporal lobar degeneration and frontotemporal dementia (Pengas et al. 2010; Bird et al. 2010; Tu et al. 2015).

Spatial disorientation is one of the earliest signs of AD (Pai and Jacobs 2004), and recent research even suggests that adults at a higher genetic risk of AD show signs of navigation deficits decades before they may develop AD (Kunz et al. 2015; Coughlan et al. 2019). Severe AD-related navigation deficits have been reported already in the earliest stages of AD for all types of navigation tasks, including path integration, route learning and cognitive mapping (see (Serino et al. 2014) for a systematic review).

While AD affects both egocentric and allocentric navigation tasks, these effects seem more pronounced in spatial tasks that require a hippocampal-dependent allocentric memory, such as locating object or landmarks relative to the environment, rather than in tasks which can be solved with egocentric parietal memory in which object locations are remembered relative to the navigator (Kalova et al. 2005; Burgess et al. 2006). The prevalence of allocentric impairments in AD is likely due to AD-related neurodegeneration, which begins in the medial temporal lobe and related structures (including the hippocampus) (Alafuzoff et al. 2008). Finally, AD has also been suggested to affect the translation between egocentric and allocentric representations, which is facilitated by the retrosplenial cortex (Serino et al. 2014).

It is also important to point out that AD-related impairments in navigation abilities cannot ‘simply’ be explained by generic learning or memory deficits (Cushman et al. 2008). Instead, these deficits are the result of the substantial overlap between the brain areas involved in navigation and those among the earliest to be affected by AD (Lester et al. 2017).

## Design factors affecting navigation in complex buildings

### Navigability

In environmental psychology, navigability is related to good design for navigation, a key aspect of environmental quality (Zimring 1982, 1990). Navigability is defined as the extent to which various destinations can be reached with reasonable effort and within a reasonable time (Carpman and Grant 2002). Certain environmental features can generate wayfinding difficulties, particularly in large buildings or environments, that cause a waste of time and money (Zimring 1990), add to users’ cognitive and physical effort (Carlson et al. 2010), and make users impatient with, or even hostile towards public buildings that are difficult to navigate (Berkeley 1973; Dixon 1968; McKean 1972). The emotional aspects of environments are considered particularly important in health care facilities used by people who may be anxious about their health issues or submitting to clinical examinations (Devlin 2014). Negative emotions towards buildings that are difficult to navigate can also develop in more ‘neutral’ buildings, such as libraries (Carlson et al. 2010) and university campuses (Abu-Ghazzeh 1996). In the last fifty years, many studies have analysed different types of building and suggested guidelines on good design for navigation (Devlin 2014).

Kaplan (1973, 1976) was one of the first authors to examine what factors facilitate indoor wayfinding, highlighting the importance of distinctive visual landmarks and a comprehensible layout of the system of paths through an environment. Garling et al. (1986) identified three architectural factors that affect the navigability of environments: degree of differentiation; visual access; and spatial layout complexity.

### Differentiation

An environment’s degree of differentiation, or uniformity (Evans et al. 1980), is related to visual or interior design features, as well as spatial features. Visual differentiation has to do with how readily different elements of a building, such as corridors, floors, etc., are visually distinguishable. Varying sizes, shapes, architectural styles and colours within a building can have positive effects on its navigability (Gärling et al. 1986). The degree of visual uniformity or differentiation affects not only newcomers or visitors unfamiliar with the environment, but also those who use it regularly and are familiar with the environment (Gärling et al. 1986).

Spatial differentiation also plays an important part in wayfinding. Low levels of spatial differentiation—due to the repetition of similar elements or symmetrical structures, for instance—can give rise to problems with orientation (Baskaya et al. 2004). One reason could be that repeated or symmetrical structures make it harder to understand which part of a building we are in. This hypothesis is inconsistent, however, with studies showing that buildings designed along gestalt principles (such as symmetry) were judged easier to navigate (Weisman 1981; Canter 1974). So, it might be that symmetrical and harmonious structures facilitate the construction of cognitive maps (Kaplan 1973). This contradiction highlights how important it is to strike the right balance between diversity and uniformity: a ‘good’ symmetrical building that is likely to be easier for users to represent mentally should also contain diverse visual features that help them to locate themselves within it.

The above-mentioned elements that support indoor navigability are universal, i.e. they are applicable to a broad range of buildings—including health care environments (Devlin 2014), housing for the elderly (Devlin 1980), libraries (Carlson et al. 2010) and university campuses (Abu-Ghazzeh 1996).

### Visual access and layout complexity

Abu-Ghazzeh (1996) interviewed university students about design elements that contributed to the wayfinding difficulties they experienced on the university campus. In line with the research discussed above, the students mentioned the degree of uniformity, but also visual access and layout complexity. A limited visual access resulted in difficulties with recognising and locating distant destinations, and made it difficult for people to see where they were headed. Complex layouts with too many route options, too many choice points along corridors, and too many corners (which also affect visual access Gärling et al. 1986) also reportedly affected the students’ navigation performance. This is in line with earlier research showing that floor plan complexity negatively influences wayfinding performance in built environments (O’Neill 1992).

## Design factors that affect navigation in older adults and people with dementia: working towards improved ageing- and dementia-friendly design guidelines

As discussed above, navigation abilities decline in typical ageing and in people with AD, and novel, unfamiliar environments become particularly difficult to navigate. When older adults with and without AD decide to move into sheltered housing or care homes, it is therefore of paramount importance to ensure that these environments are designed to compensate for their residents’ declining orientation skills. We here use empirical evidence and neuropsychological theories to develop improved ageing- and dementia-friendly design principles that aim to minimise spatial disorientation in the built environment. It is important to note at this point that care environments differ vastly in terms of their size, architecture, available resources, and freedom to implement changes. We therefore aim to provide generic design principles rather than specific solutions. These design principles can then be translated into bespoke solutions for specific environments.

### Architectural design: layout

When developing environments for people with declining navigation abilities, the design should consider all the above-mentioned issues, aiming for residential buildings with simple layouts (Marquardt and Schmieg 2009), excellent visual access (Gärling et al. 1986), little uniformity (Evans et al. 1980), a clear signage system (Carpman and Grant 2002), and the presence of landmarks (Ishikawa and Nakamura 2012), for an overview see (Marquardt 2011). The most effective layouts for care homes are small-scale units where everything a resident needs is always in view (visual access) from wherever they are within the facility (Caspi 2014). The ‘Green House’ in Mississippi in which all en-suite bedrooms open directly onto a communal ‘hearth’ space, enabling residents to navigate between their own rooms and the sitting, dining, and activity areas with ease, illustrates this concept well (Rabig et al. 2006). The reason why small-scale units with good visual access reduce spatial disorientation is that this layout makes spatial memory and recall, as well as wayfinding decisions, redundant (Marquardt and Schmieg 2009).

While it is clear that small units with good visual access are the best solution for people with declining navigation abilities, the reality is that the new homes that opened in UK in the 12 months to April 2018 were twice the size of those that closed in the same period, with an average of 60 beds (KnightFranks 2018). As the majority of care homes are privately run (Macdonald and Cooper 2007), limited space and financial considerations may require them to maximise the number of residents for the space available. Structural and financial issues can then result in architectural choices that confuse and disorient residents with dementia. It is therefore crucial for interior design solutions to focus on residents’ residual navigation abilities to minimise spatial disorientation.

### Places and corridors

#### Places should be distinguishable and have meaning

The efficiency of their spatial navigation depends on people’s ability to immediately recognise the place they are in or navigating to. Many of the auditing tools that address the ‘dementia-friendliness’ of an environment, e.g. the EHE assessment tool by the King's Fund (The King’s Fund 2013), recommend making private bedrooms and bedroom doors highly distinguishable to help with recognition. Empirical evidence (Davis and Weisbeck 2016 for a review) highlights that this can be done by placing a portrait of the resident and a name sign on their doors (Nolan et al. 2001), or with memory boxes that contain pictures and other personal objects (Nolan et al. 2002).

Communal spaces in residential developments or care homes often feature more than one access point which makes them decision points in the context of navigation. Given the importance of decision points for navigation (Aginsky et al. 1997), it makes sense for dementia-friendly design guidelines to place much emphasis on making places easily recognisable and meaningful (The King’s Fund 2013), by using landmarks, for example. The same holds for large places or areas within the built environment, where designers have more options, however. For example, differently designed or coloured furniture, different colour schemes, or different wallpaper can be used so that different places are clearly distinguishable (Davis et al. 2008). Places should also be meaningful and could even be given names that reflect their function, which will also help residents to use them for navigation and orientation.

#### Corridors should be distinguishable

In large built environments, corridors often look very similar, and this is the most frequently mentioned factor causing disorientation in people with memory problems or dementia. This quote demonstrates this point well: ‘*You can get completely disorientated and the reason is because all the corridors are the same. You don’t know which one you’re on, or what level you’re on really until you look at the little messages on the side … (Colin)*’ (O'Malley et al. 2017). One of the main reasons why corridors that look similar pose particular difficulties for older adults with MCI and AD is that their ability to track their position in space on the basis of ego-motion information is severely diminished (i.e. impaired path integration (Mokrisova et al. 2016). In other words, people with dementia (and those with MCI or AD in particular) need landmarks or other environmental cues to disambiguate visually similar situations. As discussed earlier, corridors with clearly different design features that are visible from decision points could also serve as beacons, helping residents to use simple landmark-based navigation strategies that minimise memory load.

There are various ways in which corridors can be designed to make them more distinguishable. O’Malley et al. (2017) interviewed people with memory problems and dementia about their design preferences regarding navigation. They found that participants complained about boring, repetitive, impersonal and nondescript pictures on walls in corridors. In line with the previously discussed design suggestions for landmarks, they preferred bright pictures and those that had meaning for them. This could be achieved in care environments with many local residents, for example, by using pictures of easily recognisable local landmarks.

Other ways to make corridors more distinguishable and help with orientation and navigation include using different themes (e.g. an ocean corridor, a forest corridor), different colours for walls and doors, different-coloured frames for pictures on the walls, or differently designed entrances to different corridors so that they are easy to differentiate.

The use of colours to differentiate areas along corridors or to separate floors was the design suggestion that people with dementia most frequently mentioned (O'Malley et al. 2017). In selecting colour schemes, it makes sense to give people with dementia a voice, as staff and care home residents often have different preferences (Godwin 2014). We discuss colour choices in design for dementia in more detail below.

### Landmarks

Landmarks and signage are probably the most important interior design elements that can help with orientation and navigation in large complex environments, and people with dementia continue to use them to get about (O’Malley et al. 2017; Sheehan et al. 2006). As discussed earlier, landmarks should be unique, salient, persistent and informative. This is particularly important for older adults and those living with dementia, who may experience deterioration in physical, mental and sensory abilities, resulting in poorer perception and cognition, reduced mobility, and visual impairment. Below we make suggestions about how such landmark properties could be implemented.

#### Landmarks should be brightly coloured and feature high contrast

Objects that serve as landmarks need to be remembered and recognised. Older adults and people with dementia remember and recognise brightly coloured, high-contrast objects more easily than less-striking objects in pastel shades (Cernin et al. 2003). People with dementia reportedly use colour cues, such as different-coloured doors, to aid navigation (Gibson et al. 2004). It should be noted, however, that they are also particularly susceptible to being distracted by colour cues that are inappropriately placed or irrelevant (Wood et al. 1997). After learning a novel route, older adults were also found to mention salient objects more than turns as the most useful sources of information when learning novel routes, even when the objects concerned were not in navigationally relevant locations (Lipman 1991). This suggests that, if colour and contrast is used to facilitate wayfinding, they need to be used with caution and mainly for features that are meant to be used as landmarks or navigational cues. Brightly coloured objects in locations that are irrelevant for navigation purposes should be avoided.

#### Landmarks should be nameable

While it is important for landmarks to be visually distinct, people navigating a route often use verbal codes to memorise landmarks and directions (Meilinger et al. 2008; Grzeschik et al. 2018). For example, when learning an unfamiliar route, they may recall a particular change of direction as ‘turn right at the wall clock’; in this case, the wall clock serves as a landmark. Language and word recall problems are common in dementia, however, particularly in frontotemporal and semantic dementia (for an overview, see Klimova and Kuca 2016). To counter the increasing word-finding problems associated with dementia (Rohrer et al. 2008) and to support the verbal encoding of landmarks, it is crucial to choose landmarks or cues that are concrete, rather than abstract (such as an abstract painting), and therefore easy to name.

#### Landmarks should be both visually and verbally distinguishable

As discussed above, landmarks need to be unique in order to identify a place unambiguously (Stankiewicz and Kalia 2007a). Since they can be encoded both visually and verbally, it is important to ensure that they are distinguishable in both domains. For example, the visual appearance of two paintings of sunflowers can be very different, but both depict the same object. People with dementia who use pictures and paintings in corridors as navigation aids (O’Malley et al. 2017) may verbally encode both these landmarks as ‘the sunflower painting’. This could lead to confusion and disorientation, because the same mental representation would be associated with two different places.

#### Landmarks should to be placed at navigationally relevant locations

Generally, people navigating along a route remember landmarks that are located at decision points better than those located elsewhere along the route (Aginsky et al. 1997). People also pay more attention to the navigation task when approaching points where a decision is needed (Hartmeyer et al. 2017), and the objects they encounter at these decision points recruit a different neural network from those seen at other locations (Janzen and Tourennout 2004).

In indoor environments, decision points are typically either junctions where corridors meet or larger open areas that can be accessed from different directions. When learning a way through unfamiliar environment, navigators typically associate a direction of movement with a decision point such as ‘turn right at the reception area’ (Waller and Lippa 2007; O'Malley et al. 2017). While the ability to form these associations declines in both healthy ageing (Zhong and Moffat 2016) and atypical ageing (O'Malley et al. 2018), older adults still retain some ability to use this ‘associative cue’ strategy for navigation. It is therefore important to design decision points so that they are easily recognisable and distinguishable, and to use landmark cues that are easily nameable. This has implications for design—not only for landmark objects, but also for spaces, which should be given meaning (and sensible names if possible) to support navigation.

#### Landmarks should be placed so that they can serve as beacons

Recent research has shown that older adults rely heavily on beacons to support navigation (Wiener et al. 2013). Beacons are environmental cues or landmarks that we can see from decision points, and moving towards a given beacon brings one closer to the destination (Waller and Lippa 2007). Beacon-based navigation strategies are less memory demanding than associative cue strategies because we do not need to associate a direction of movement with the landmark—an ability that declines as we grow older (O'Malley et al. 2018; Zhong and Moffat 2016). People with MCI and AD show very similar levels of performance to healthily ageing adults when it comes to recognising objects encountered while learning a route (Cushman et al. 2008), which is the only knowledge required if the objects are located so as to act as beacons. In indoor environments, beacons can be provided simply by adding distinctive objects (as discussed above) in corridors. When residents arrive at a decision point, they can ‘simply’ look around and select the corridor with the beacon that guides them towards their destination.

### Signage and maps

Signage and maps can be used to overcome many orientation and navigation problems. Signage and maps essentially externalise spatial knowledge, decision-making and spatial planning, and can therefore compensate for declining cognitive abilities (Passini et al. 2000). Importantly, people with dementia continue to use signage for orientation and wayfinding (Sheehan et al. 2006).

#### Signage

Despite the ubiquity and importance of effective signage systems in public spaces, surprisingly few studies have addressed the impact of different signage designs and systems on wayfinding performance. While locally based wayfinding signage evaluations can produce very effective solutions for individual sites, the information gathered to guide changes to existing signage systems often depends more on users’ subjective perceptions than on objective measurements of their orientation and navigation performance. We therefore should not take for granted that existing signage design guidelines are generally applicable (e.g. Carpman and Grant 2002; Arthur and Passini 1992; Cooper 2010; Devlin and Bernstein 1997; Tufte 1990; Zhang et al. 2010).

There are, however, a number of generic design suggestions for dementia-friendly signage that address people’s ability to recognise and understand these navigation aids:

##### Signage should contain both icons and text

Problems with language and reading comprehension are common in dementia (Klimova and Kuca 2016). Signage should therefore not rely on textual information alone, but also contain pictures or icons. Wayfinding signage that relies only on iconic information alone has also proved difficult to understand, particularly for those with cognitive impairments, but providing icons or pictorial information along with text on the same sign can greatly improve matters (Scialfa et al. 2008). Signs should also be simple and uncluttered, preferably containing only one or two words (Scialfa et al. 2008) and, to compensate for the visual impairments associated with dementia, there should be a clear contrast between the text and the background colour.

##### Signage should use familiar icons or pictorial information

Comprehension of signs and icons is negatively affected by dementia of the Alzheimer’s type (Scialfa et al. 2008). As mentioned above, signs can be made easier to understand by providing both text and images together. Importantly though, familiarity is an important predictor of icon and symbol comprehension (Hancock et al. 2004, 2005), which suggests that icons or pictorial information used for signage should be familiar to a care home residents. Since familiarity is based on memory, which declines in dementia, it is also important for the icons used in signage for people with dementia to be representational rather than abstract.

##### Signage should be placed where it can be seen

Older adults tend to look down whilst navigating (Namazi and Johnson 1991), possibly to monitor their locomotion, look out for tripping hazards, and reduce the risk of falls, which increases with ageing, and even more with dementia (Liu-Ambrose et al. 2008). This has implications for the placement of landmarks and signage: to ensure they are not missed, they should be placed not high up on ceilings or walls, but at eye level or even lower. In fact, Namazi and Johnson (1991) found that a series of directional arrows on the floor with the word ‘toilet’ produced the most successful use of toilets in a dementia care home.

##### Signage and homely environments

Despite the efficiency of good signage systems, most of the care home residents interviewed in a recent study said they preferred more personal environmental cues or features, such as relevant pictures and memorable spaces, over signage or maps (O’Malley et al. 2017). The authors concluded that this reflects residents’ desire to live in environments that are more homely and less like institutions (schools, hospitals, and so on), which typically feature signage and you-are-here (YAH) maps. An important challenge for future research is therefore to design wayfinding signage and maps that comply with the above-outlined design suggestions without detracting from the homely look and feel of an environment.

#### You-are-here maps

In indoor environments, YAH maps are typically mounted on a wall and show the floor plan of an environment. They also feature a symbol that indicates the position of the map (and therefore of the person standing in front of it) in the environment they depict. It is important for YAH maps to be oriented so that ‘up’ on the map is aligned with straight ahead in the real environment (McKenzie and Klippel 2016). This minimises the cognitive effort needed—or, to be more specific, the need for mental rotation, another spatial ability affected by ageing (Zhao et al. 2019)—to transfer the information on the map to the real world. There are well-established design principles for YAH maps that cover aspects such as alignment, placement, correspondence and visual clutter, which are discussed in more detail in Klippel et al. (2006).

##### YAH maps may not be useful navigation aids for people with dementia

There is currently only a handful of neuropsychological studies on map usage by older adults and people with dementia, but the available evidence suggests that maps in general, and even YAH maps, are of little use to them. For example, adults with AD attending a navigational training programme at a care home did not find maps helpful. They often discarded them, preferring to use landmarks such as the nurses’ station instead (McGilton et al. 2003). Lanza et al. (2014) likewise found that maps did not aid navigation in patients with mild-to-moderate AD.

### Colour and contrast as design features to minimise spatial disorientation

We have already mentioned colour as a design feature that can help to differentiate between different parts of buildings, or between different corridors, and this begs the question of whether some colours are particularly suitable as design features. Generally speaking, colours have long been thought to affect humans, but there is only limited evidence to suggest that particular colours prompt certain health outcomes, emotions and/or behaviours (Tofle et al. 2004). For example, there seems to be no significant relationship between wall colours in hospitals and patients’ anxiety levels, length of stay, or requests for pain medication (Edge 2003).

It should be noted at this point that AD affects people’s ability to discriminate between colours, particularly in the blue and green range, and less so in the red and yellow range (Wijk and Sivik 1995). While personal preferences for certain colours seem to remain stable despite the condition, colour naming deteriorates as AD progresses (Wijk and Sivik 1995). Colour naming and being able to make correct choices about colour becomes particularly problematic in patients with semantic dementia, which appears to be caused by the breakdown of conceptual knowledge (Rogers et al. 2007). Several studies have also shown that people with dementia are less sensitive to contrast, which has to be stronger for them to notice it (Bassi et al. 1993; Cronin-Golomb et al. 1991; Crow et al. 2003).

Space perception (i.e. a sense of spaciousness or confinement) is affected by the brightness or darkness of a colour, rather than by a specific colour or shade. A sense of spaciousness can be enhanced by lighter-coloured walls and less-contrasting furnishings (Tofle et al. 2004).

#### Sufficient light levels and suitable colours are important to the creation of supportive environments

The use of extra light and contrasting colours can help care home residents find their way more easily (Noell-Waggoner 2002; Netten 1989). Bright, strong colours can be effective in improving object recall in people with AD (Cernin et al. 2003), and could therefore play a crucial part in designing environmental features that can serve as landmarks.

#### Personalising bedroom colours

The fact that colour preferences seem to remain stable in people with AD (Wijk and Sivik 1995) suggests that personalising bedroom colours could be an effective way to help care home residents recognise their own room.

## Summary and conclusions

*Spatial navigation and wayfinding abilities:*

- are important for independent living and well-being and rely on an interplay of multiple sensory and cognitive mechanisms and processes (Hegarty and Waller 2005; Wolbers and Hegarty 2010);
- are likely to decline in typical and atypical ageing (Benke et al. 2014; Lester et al. 2017; Moffat 2009; Head and Isom 2010; Hilton et al. 2021; Iaria et al. 2009; Vieweg et al. 2015; Harris and Wolbers 2014; Liu 2011); the decline in spatial navigation abilities in MCI and AD patients is dramatic and is one of the earlier markers of the onset of disease (Fasano et al. 2018; Mitolo et al. 2013; Pai and Jacobs 2004; Serino et al. 2015; Kunz et al. 2015; Coughlan et al. 2019; Cushman et al. 2008);
- nevertheless, people affected by dementia can use adequate environmental cues to orient themselves (Davis and Weisbeck 2016);
- adequate landmarks are efficient in guiding navigation (Ishikawa and Nakamura 2012) (Ishikawa and Nakamura 2012) and people with dementia (O’Malley et al. 2017; Jansen-Osmann 2002; Davis and Weisbeck 2016; Sheehan et al. 2006).

*Environmental factors.*

- Can greatly affect spatial orientation in both outdoor and indoor environments (Marquardt and Schmieg 2009; Fleming 2011; Carpman and Grant 2002; Carlson et al. 2010; Devlin 2014; Davis and Weisbeck 2016);
- Their importance grows as people become more vulnerable, with greater dependence on the environment in ageing (Lawton 1977; Lawton and Simon 1968; Fornara et al. 2019);
- Are only marginally considered in the design guidelines and auditing tools that address the ‘dementia-friendliness’ of an environment (O'Malley et al. 2017).

*Design factors affecting navigation in complex buildings.*

- Visual and spatial differentiation: varying sizes, shapes, architectural styles and colours within a building can have positive effects on its navigability (Abu-Ghazzeh 1996; Gärling et al. 1986; Evans et al. 1980; Baskaya et al. 2004; Marquardt 2011);
- Visual access: limited visual access results in difficulties with recognising and locating distant destinations (Abu-Ghazzeh 1996; Gärling et al. 1986; Caspi 2014);
- Layout complexity: complex layouts with too many route options, too many choice points along corridors, and too many corners negatively affect navigation performance (Marquardt and Schmieg 2009; Abu-Ghazzeh 1996; Gärling et al. 1986; O’Neill 1992; Marquardt 2011).

*Design principles.*

The following design recommendations can support navigation in residential/care environments:

Layout and Corridors.

- Small-scale units reduce spatial disorientation (Marquardt and Schmieg 2009; Marquardt 2011) if they feature: few route options (Marquardt and Schmieg 2009); short corridors (O'Malley et al. 2017); symmetrical layouts (Weisman 1981; Canter 1974); good visual access (Gärling et al. 1986); and good visual differentiation at all levels: doors, private and common rooms, corridors, floors, etc. (Nolan et al. 2001, 2002; Davis et al. 2008).

Landmarks.

- Any object at the right location and with the right characteristics can be used as a landmark (Richter and Winter 2014; Ishikawa and Nakamura 2012)
- Good landmarks are: unique (Stankiewicz and Kalia 2007a; Viaene et al. 2014; Strickrodt et al. 1936); salient (Stankiewicz and Kalia 2007a; Ishikawa and Nakamura 2012); persistent (Stankiewicz and Kalia 2007a); informative (Stankiewicz and Kalia 2007a; Waller and Lippa 2007; Wiener et al. 2013; Chan et al. 2012); and located at decision points (Nothegger et al. 2004; Raubal and Winter 2002; Jansen-Osmann 2002).
- Landmarks should: have bright colours and high colour contrast (Cernin et al. 2003; Gibson et al. 2004); be concrete (Rohrer et al. 2008), nameable (Meilinger et al. 2008; Grzeschik et al. 2018), visually and verbally distinguishable (O’Malley et al. 2017; O'Malley et al. 2017), and placed at navigationally relevant locations (Cushman et al. 2008; Aginsky et al. 1997; O'Malley et al. 2017; Hartmeyer et al. 2017; Janzen and Tourennout 2004).

Signage and maps.

- Signage is useful to guide navigation (Carpman and Grant 2002; Sheehan et al. 2006; Passini et al. 2000; Arthur and Passini 1992; Cooper 2010; Devlin and Bernstein 1997; Tufte 1990; Zhang et al. 2010), but signage can diminish the sense of being at home (O'Malley et al. 2017). Maps are rarely useful to support navigation in people with dementia (Lanza et al. 2014).
- Good signage: combines icons with text (Scialfa et al. 2008); makes use of familiar icons (Hancock et al. 2004, 2005); is located at eye level or lower (Namazi and Johnson 1991).

Navigation abilities, which are important for independence and well-being, decline in (a)typical ageing. It is therefore crucial for residential developments and care environments to be designed so that support navigation. The aim of this paper was to discuss design principles for built environments that can compensate for declining orientation and navigation abilities in people with dementia. There are limits to this approach, of course, be it because changes cannot be implemented in environments or because navigation impairments are too severe. In such cases, navigation and orientation can also be supported by the use of emerging assistive technology (Maus et al. 2016). While it is beyond the scope of the present article to discuss design principles for assistive technology, such as smartphone-based navigation assistance, it is important to stress that this technology needs to be designed so that it is dementia-friendly, i.e. so that it can be used easily and intuitively by people with dementia.

In this article, we considered studies from (neuro-)psychology, environmental psychology, architecture and usability research to highlight evidence and mechanisms that underpin existing as well as novel design principles that can help to minimise spatial disorientation. Importantly, we considered interactionist models of ageing (Kahana 1982; Lawton 1977) showing that the concurrent analyses of environmental and individual factors, and their interaction can provide insight on situated behaviours. We believe that such an evidence-driven approach to the generation of design principles is crucial to improving the navigability of the built environment, particularly for people with declining navigation abilities. Overall, our review underlines the important role that design plays in minimising spatial disorientation in residential care and nursing homes and the association between the maintenance of wayfinding skills and quality of life. Of course, other factors contribute to the well-being of people with dementia and their family carers: from the use of space in the environment, the presence or lack of outside space, and, last but not least, type of caring and assistance by the staff (Innes et al. 2011). We believe that effectively improving the lifes of older people with and without dementia requires an interdisciplinary approach and the joint effort of research in different disciplines.
